# The Role of Extracellular Matrix Components in the Spreading of Pathological Protein Aggregates

**DOI:** 10.3389/fncel.2022.844211

**Published:** 2022-04-29

**Authors:** Edoardo Moretto, Skye Stuart, Sunaina Surana, Jose Norberto S. Vargas, Giampietro Schiavo

**Affiliations:** ^1^Institute of Neuroscience, National Research Council, CNR, Milan, Italy; ^2^UK Dementia Research Institute, University College London, London, United Kingdom; ^3^Department of Neuromuscular Diseases, Queen Square Institute of Neurology, University College London, London, United Kingdom; ^4^UCL Queen Square Motor Neuron Disease Centre, University College London, London, United Kingdom

**Keywords:** tau, alpha synuclein, amyloid beta, TDP-43, huntingtin, extracellular matrix, HSPG, proteases

## Abstract

Several neurodegenerative diseases are characterized by the accumulation of aggregated misfolded proteins. These pathological agents have been suggested to propagate in the brain *via* mechanisms similar to that observed for the prion protein, where a misfolded variant is transferred from an affected brain region to a healthy one, thereby inducing the misfolding and/or aggregation of correctly folded copies. This process has been characterized for several proteins, such as α-synuclein, tau, amyloid beta (Aβ) and less extensively for huntingtin and TDP-43. α-synuclein, tau, TDP-43 and huntingtin are intracellular proteins, and their aggregates are located in the cytosol or nucleus of neurons. They have been shown to spread between cells and this event occurs, at least partially, via secretion of these protein aggregates in the extracellular space followed by re-uptake. Conversely, Aβ aggregates are found mainly extracellularly, and their spreading occurs in the extracellular space between brain regions. Due to the inherent nature of their spreading modalities, these proteins are exposed to components of the extracellular matrix (ECM), including glycans, proteases and core matrix proteins. These ECM components can interact with or process pathological misfolded proteins, potentially changing their properties and thus regulating their spreading capabilities. Here, we present an overview of the documented roles of ECM components in the spreading of pathological protein aggregates in neurodegenerative diseases with the objective of identifying the current gaps in knowledge and stimulating further research in the field. This could potentially lead to the identification of druggable targets to slow down the spreading and/or progression of these pathologies.

## Introduction

Most neurodegenerative diseases are characterized by the accumulation of misfolded protein aggregates. These include tau in tauopathies, α-synuclein in synucleinopathies, such as Parkinson’s disease (PD), TDP-43 in frontotemporal dementia (FTD) and amyotrophic lateral sclerosis (ALS), huntingtin in Hungtinton’s disease (HD) and amyloid beta (Aβ) in Alzheimer’s disease (AD) (Ross and Poirier, 2004). These aggregates exert varying degrees of toxicity on neurons and glial cells, ultimately driving their degeneration. Although each of these diseases originates in and affects different regions in the brain, all show some level of spreading of pathology over time. Each disease also exhibits characteristic rates and routes of distribution of pathological protein aggregates to other brain regions (reviewed in Brettschneider et al., 2015). For example, tau aggregation in AD initiates in the locus coeruleus and entorhinal cortex and subsequently spreads to the hippocampus, reaching the neocortex only at later stages of the disease. Aβ plaques instead are first observed in the neocortex, only afterward reaching deeper brain structures in later stages of AD. α-synuclein deposits, on the other hand, are first observed in the olfactory bulb and the dorsal motor nucleus of the vagus nerve, subsequently spreading to the midbrain and later, to the neocortex.

These pathological aggregates have the ability to induce downstream aggregation of natively folded proteins similarly to the prion protein (Vaquer-Alicea and Diamond, 2019). This finding, along with the observation that patterns of diffusion across brain regions are conserved between patients, suggests that some form of seed exist, which have the ability to travel across the brain and spread pathology. This spreading activity is distinguishable in two main classes, one involving intracellular proteins and the other, extracellular aggregates. Tau, α-synuclein, TDP-43 and huntingtin all have intracellular localization, and their aggregates also form intracellularly, whereas Aβ aggregates are found predominantly extracellularly. For intracellular proteins, aggregation is believed to start in a subset of cells from which seeds are then released, either by active secretion or passively due to cell death. Such proteopathic seeds would then be endocytosed by other cells and act as a template for the misfolding of endogenous proteins, thus causing further aggregation. Extracellular aggregation-prone Aβ, on the other hand, could move to different brain regions by simple diffusion in extracellular fluids and nucleate aggregation of locally generated Aβ.

These pathological proteins have been suggested to be present in the extracellular space in free forms, although at least some of them have also been found in extracellular vesicles (e.g., exosomes) or in tunneling nanotubes (Lee et al., 2011). It is thus clear that both classes of proteins will, at some point, be in the extracellular space and therefore enter into contact with components of the extracellular matrix (ECM). The ECM is a ubiquitous and complex protein network present in the space between cells of solid tissues, including the nervous system (Ruoslahti, 1996; Novak and Kaye, 2000; Krishnaswamy et al., 2019). Primarily consisting of laminins, collagens, glycoproteins and proteoglycans, the ECM undergoes regulated remodeling by virtue of extracellular proteases. All of these ECM components are secreted by neurons as well as glia and, in addition to providing physical support for these cells, play pivotal roles in regulating cell division, differentiation and migration, among other functions (Dityatev and Schachner, 2003; Dityatev et al., 2010). Since ECM components form an extracellular meshwork, they regulate the diffusion of molecules in the brain. Thus, rather unsurprisingly, components of the ECM can modulate the properties and spreading of these proteopathic seeds in neurodegenerative diseases. Interestingly, a growing body of evidence suggests that the levels of ECM components are severely affected in several neurodegenerative diseases, such as AD, PD, and ALS (Wong and Venkatachalam, 2019; NeuroLINCS Consortium et al., 2021; Downs et al., 2022; Johnson et al., 2022).

In this review, we summarize the known roles of ECM components in the spreading process, with the largest body of evidence existing for proteoglycans and extracellular proteases. Proteoglycans, which are either found in secreted form or bound to the plasma membrane, are glycosylated proteins which are post-translationally modified by the addition of glycosaminoglycans. The most abundant proteoglycans are heparan sulfate proteoglycans (HSPGs) and chondroitin sulfate proteoglycans (CSPGs), both of which are involved in different steps of the spreading process, including endocytosis of seeds, promoting aggregation and protecting aggregates from degradation (Holmes et al., 2013; Maïza et al., 2018). In addition to typical ECM proteases such as zinc-dependent matrix metalloproteinases (MMPs), additional proteases have been found in the extracellular space and altogether regulate ECM functions (Wang et al., 2008; Krishnaswamy et al., 2019). As detailed below, many of these enzymes cleave proteins that aggregate in neurodegenerative diseases, generating fragments with reported pro- and anti-aggregating effects. Although some of these proteases, such as calpains and cathepsins, mainly localize intracellularly and their activity on proteopathic proteins has been investigated in this context, their presence has also been reported in the extracellular space. Here, we discuss the possibility that cathepsins and calpains retain their activity on protein aggregates in the extracellular milieu. Emerging evidence suggests that this area of research requires further attention, especially as understanding the regulatory roles of ECM components on spreading may identify potential therapeutic targets that could reduce the progression of these devastating diseases.

## Tau

Tau is a highly expressed neuronal protein that, through its microtubule binding region (MTBR), binds to and stabilizes axonal microtubules. The MTBR tends to form β-sheets which drive protein aggregation (Wang and Mandelkow, 2016). In pathological conditions, collectively named tauopathies, tau loses its affinity for microtubules, becomes hyper-phosphorylated, and aggregates into oligomers, fibrils and neurofibrillary tangles (NFT) (Braak et al., 1994). Pathological tau aggregation and propagation follows a characteristic spatiotemporal sequence between functionally connected brain regions (Braak and Braak, 1991). This has led to the hypothesis that pathological tau is secreted by a “donor” neuron into the extracellular space before being internalized by “acceptor” neurons (Holmes and Diamond, 2014). This hypothesis implies that tau is exposed to the extracellular environment where different proteases and ECM components could affect its ability to propagate pathology.

### Proteoglycans

**FIGURE 1:**
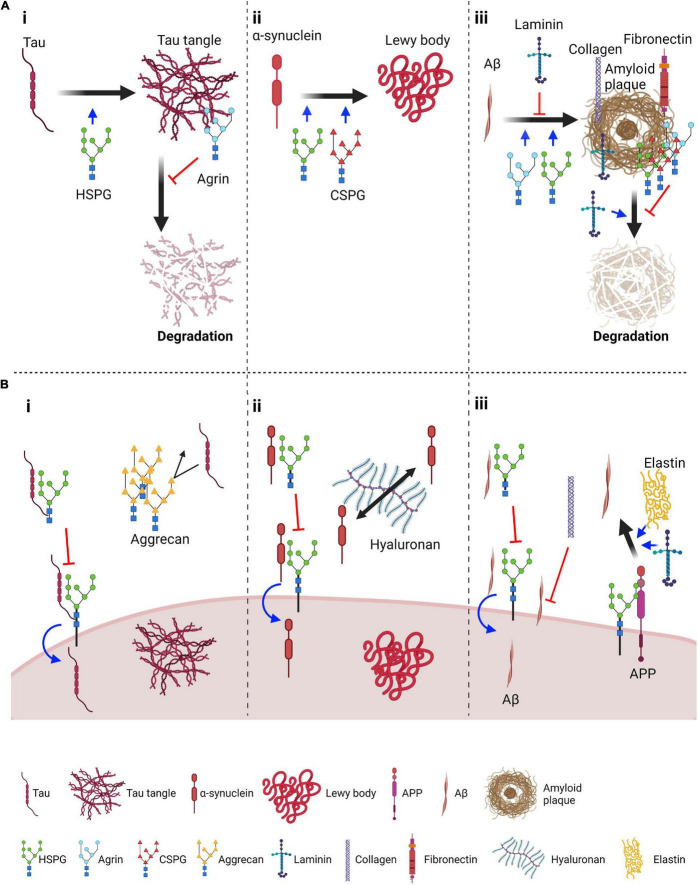
Roles of proteoglycans and ECM components in the aggregation and spreading of tau, α-synuclein and amyloid β. **(A)** Sugg*e*sted roles of proteoglycans and core ECM components in aggregation and degradation of pathological protein aggregates: (i) Tau panel: HSPGs promote tau aggregation; Agrin, an extracellular HSPG, localizes on neurofibrillary tangles and prevents their degradation; (ii) α-synuclein panel: HSPGs and CSPGs promote aggregation of α-synuclein; (iii) Aβ panel: HSPGs and agrin can promote Aβ aggregation whereas laminin has been suggested to impair this process. Several proteoglycans, including HSPGs, agrin and CSPGs, and ECM components such as laminin, collagen and fibronectin associate with amyloid plaques. Of these, laminin enhances plaque degradation whereas proteoglycans inhibit this process. **(B)** Suggested role of proteoglycans and ECM components in endocytosis and spreading: (i) Tau panel: HSPGs on the plasma membrane favor tau seed endocytosis. In contrast, if tau is bound to extracellular HSPGs, its interaction with surface HSPGs might be inhibited, thus reducing tau endocytosis. Aggrecan perineuronal nets have been shown to reduce propagation of tau pathology, possibly by acting as a barrier; (ii) α-synuclein endocytosis is also promoted by HSPGs and similarly extracellular HSPGs can inhibit endocytosis. In the extracellular space, hyaluronan was found to promote spreading of α-synuclein pathology; (iii) Aβ panel: as for tau and α-synuclein, transmembrane HSPGs can promote internalization of Aβ, and this might be inhibited by extracellular interactions. Collagen has been found to reduce the interaction of Aβ peptides with the cell surface. HSPGs have also been found to promote Aβ production from APP, a process that is also promoted by ECM components such as elastin and laminin. Figure was prepared with Biorender (Biorender.com).

Heparan sulfate proteoglycans play an important role in the spreading of tau pathology (Figures 1Ai,Bi). HSPGs are proteoglycans characterized by one or more heparan sulfate (HS) groups linked to a protein core. HS is composed of disaccharide chains consisting mainly of glucuronic acid and *N*-acetyl-D-glucosamine (Sarrazin et al., 2011). HSPGs exist in different classes: transmembrane HSPGs, including glypicans and syndecans; serglycins, found in extracellular vesicles; and secreted HSPGs, such as perlecans and agrins (Sarrazin et al., 2011; Condomitti and de Wit, 2018). HSPGs bind to tau and have been shown to act at all stages of its spreading: secretion into the extracellular space (Zehe et al., 2006; Katsinelos et al., 2018), cellular uptake (Holmes et al., 2013; De La-Rocque et al., 2021) and self-assembly into higher-order states (Zhao et al., 2021). Tau binds to heparin, a densely sulfated form of HSPG, through both the N-terminus and the MTBR (Goedert et al., 1996). The sulfate moieties on HSPGs appear to be crucial for tau binding, requiring both 3-*O*- and 6-*O*-sulfation (Zhao et al., 2017, 2020; Stopschinski et al., 2018). Heparin induces a conformational change in the MTBR and its flanking region that exposes previously masked tau phosphorylation sites and can induce oligomerization (Paudel and Li, 1999; Sibille et al., 2006; Elbaum-Garfinkle and Rhoades, 2012). However, heparin and related molecules inhibit tau uptake (Zhao et al., 2017; Weisová et al., 2019; Puangmalai et al., 2020), thus preventing tau fibrils from driving intracellular aggregation (Holmes et al., 2013), as shown by the heparin mimetic F6 (Holmes et al., 2013). These results suggest that an excess of extracellular HSPGs could have similar beneficial effects. However, a synthetic heparinoid with nanomolar affinity for tau failed to show any effect on tau pathology after chronic administration *in vivo* (Stopschinski et al., 2020). It is important to note that most studies have concluded that cell surface-bound HSPGs are involved in tau transfer without assessing the possible contribution of extracellular HSPGs.

Agrin, a major extracellular HSPG, accumulates in AD brains and more specifically, in NFTs (Verbeek et al., 1999; Kroger and Schroder, 2002; Smith and Hilgenberg, 2002; Del Campo Milan et al., 2015). Since agrin contains protease-inhibiting domains, it has been suggested to protect protein aggregates against extracellular proteolysis, leading to the accumulation of these deposits (Verbeek et al., 1999). In addition, agrin may participate in tangle formation because sulfated glycosaminoglycans have been shown to stimulate tau phosphorylation, thus promoting the formation of paired helical filaments (PHFs) (Goedert et al., 1996; Wang et al., 1996; Hasegawa et al., 1997). However, whether or not agrin displays these activities and how it interacts with tau tangles remains to be elucidated.

The CSPG aggrecan forms perineuronal nets predominantly ensheathing fast spiking interneurons (Morawski et al., 2012; Suttkus et al., 2014; van’t Spijker and Kwok, 2017). These nets were proposed to be neuroprotective against pathological tau, since areas with high densities of perineuronal nets in post-mortem AD brains were largely spared of tangles, even at late disease stages (Brückner et al., 1999, 2008; Diamandis et al., 2000; Morawski et al., 2010a,b). Follow-up studies confirmed that the protective action of perineuronal nets was, in fact, mainly mediated by aggrecan. Most interestingly, aggrecan was shown to generate an external barrier restricting internalization and distribution of exogenous tau in organotypic slices (Nowicka et al., 2009; Suttkus et al., 2014, 2016). Indeed, aggrecan knock-out mice presented elevated tau uptake (Suttkus et al., 2016). Based on these results, it was hypothesized that these CSPGs may act by inhibiting the interaction of tau with HSPGs. However, in a recent study, mice expressing human tau carrying the FTD-linked mutation P301L crossed with heterozygous aggrecan KO mice, displayed changes in the expression and phosphorylation of tau but unaltered distribution of tau aggregates (Schmidt et al., 2021).

### Proteases

Several proteases have been shown to cleave tau (Table 1). Multiple studies have shown elevated levels of several MMPs in tauopathies as well as demonstrated functional links between different MMPs and tau. For example, upregulated MMP-3 levels were found in the cortex of AD-like amyloidosis transgenic rat models (Pentz et al., 2021), and the concentration of MMP-3 and levels of total and phosphorylated tau positively correlate in the cerebrospinal fluid (CSF) of AD patients (Stomrud et al., 2010; Hanzel et al., 2014). Although MMP-9 is enriched in AD patients’ brains and co-localizes with tau, it is sparsely present in extracellular NFTs (Hernandes-Alejandro et al., 2020). Nübling et al. (2012) tested the effect of MMP-3 and MMP-9 on tau oligomer formation and aggregation. While MMP-3 mildly reduced tau aggregation, MMP-9 processing promoted oligomerization (Nübling et al., 2012; Wang et al., 2014). Multiple potential MMP-3 cleavage sites were identified within the MTBR of tau (Nübling et al., 2012), suggesting this protease could inhibit tau aggregation by degrading regions crucial for oligomer formation. In contrast, MMP-9 cleavage sites were mainly located either in the N-terminal region or close to the C-terminus (Nübling et al., 2012), thus sparing the MTBR and facilitating the generation of tau oligomers. More recently, docking simulations have predicted that a high-affinity complex can be formed between MMP-9 and full-length tau (Hernandes-Alejandro et al., 2020). This binding involves the catalytic domain of MMP-9, suggesting that this interaction could be initiated when MMP-9 is active. Interestingly, MMP-9 can be directly activated by MMP-3 (Ogata et al., 1992; Okada et al., 1992; Shapiro et al., 1995), implying that elevated MMP-3 levels might result in increased MMP-9 activity, indirectly facilitating tau aggregation. MMP-2 also has the capacity to cleave recombinant tau *in vitro* but fails to process its hyper-phosphorylated forms in NFTs (Terni and Ferrer, 2015). It is thus possible that the accumulation of MMP-2 around NFTs found in the entorhinal cortex at the early stages of AD (Terni and Ferrer, 2015) is a potential response aimed at eliminating the production of toxic fragments in AD brains. The cleavage sites of MMP-2 on tau, however, are yet to be determined. In conclusion, MMP-2, MMP-3 and MMP-9 show differential actions on tau aggregating behavior, suggesting their differential contribution to tau pathology.

**TABLE 1:** This table summarizes the known extracellular proteases of prion-like proteins, their region and site of cleavage, the resulting fragment and the effect of their activity on spreading and aggregation of the pathological proteins.

Tau					
Protease	Cleavage region	Cleavage site	Fragments produced	Effect on spreading/aggregation	References
MMP-2	Potentially C-terminus*	Unknown	Potentially N-terminus*	Unknown	Terni and Ferrer, 2015
MMP-3	MTBR	Multiple potential cleavage sites	Unknown	Reduces aggregation	Nübling et al., 2012
MMP-9	N- or C-terminus	Multiple potential cleavage sites	Unknown	Enhances aggregation	Nübling et al., 2012
Cathepsin D	Multiple	Phe8, Met419, Leu436, Thr427, Leu428 Additional sites detected between Asp34-Gly161, Pro200-Lys257, and Lys267-Asp358	Contains MTBR	Potential to enhance aggregation* *In vivo*, reduces neurodegeneration	Kenessey et al., 1997; Khurana et al., 2010
Cathepsin L	MTBR	Lys257	258–372	Unknown	Wang et al., 2009
	MTBR	Ile360	258–360	Enhances aggregation	Wang et al., 2009
	MTBR	Val363	258–363	Enhances aggregation	Wang et al., 2009
Cathepsin S	Unknown	Unknown	Approximately 34 and 24 kDa	Enhances aggregation	Nübling et al., 2017
Calpain-1	C-terminus	Arg242	243–441 (24 kDa)	*In vitro, a*ccelerates aggregation Enhance seeding activity	Matsumoto et al., 2015
	N-terminus to mid-domain	Lys44	45–230 (17 kDa)	*In vivo* and *in vitro*, accelerates degeneration and synaptic defects *In vitro*, reduces aggregation	Yang and Ksiezak-Reding, 1995; Amadoro et al., 2006; Park et al., 2007; Ferreira and Bigio, 2011; Reinecke et al., 2011; Lang et al., 2014; Chen et al., 2021
	N-terminus to mid-domain	Arg230	45–230 (17 kDa)	*In vitro*, neurotoxic	Park and Ferreira, 2005
	N-terminus to mid-domain	Arg230	26-230	*In vitro*, neurotoxic	Park and Ferreira, 2005
	N-terminus	Gln124	125–230	No alterations to cell health	Garg et al., 2011
Calpain-2	N-terminus to mid-domain	Arg230	*45–*230 (17 kDa)	No alterations to cell health	Garg et al., 2011
	N-terminus	Lys224	Unknown	Unknown	Cicognola et al., 2019
	N-terminus	Gln124	125–230	No alterations to cell health	Garg et al., 2011
Thrombin	Proline-rich and MTBR	Arg155, Arg209, Arg230, Lys257, and Lys340	Unknown	Unknown	Arai et al., 2005
	N-terminus	Gln124	125–230	No alterations to cell health	Garg et al., 2011

**α-synuclein**					

**Protease**	**Cleavage region**	**Cleavage site**	**Fragments produced**	**Effect on spreading/aggregation**	**References**

MMP-1	Multiple	Ala19, Lys21, Gly41, Gly47, Thr72, Gln79, Ala91, Asp98, Tyr133	Unknown	*In vitro*, enhances aggregation	Levin et al., 2009
MMP-2	Unknown	Unknown	Unknown	*In vivo*, reduces aggregation and spreading	Oh et al., 2017
MMP-3	N-terminus	Between Thr54-Glu57	Unknown	Potential to enhance aggregation*	Sung et al., 2005
	NAC domain	Ala78	Unknown	Enhances aggregation and increases toxicity	Choi et al., 2011
	NAC domain	Gly93	1-93	*In vivo*, no change in aggregation, toxicity and spreading	Choi et al., 2011
MMP-9	Unknown	Met5, Leu8, Ala19, Val66, Val70, Val74, Ala78, Gln79	Multiple	*In vitro*, reduces aggregation	Levin et al., 2009
Plasmin	N-terminus and NAC	Ala11, Thr33, Thr44, Thr59, Thr81, Asp98	Unknown	*In vitro*, reduces aggregation and spreading	Kim et al., 2012
Neurosin	NAC domain	Lys80, Lys97, Glu114, Asp121	Unknown	*In vitro and in vivo*, reduces aggregation	Tatebe et al., 2010; Pampalakis et al., 2017
Cathepsin B	Unknown	Gly14 and Ala90	Several fragments, most prominent at 10 kDa	No effect on seeding capabilities	Tsujimura et al., 2015; McGlinchey et al., 2020
Cathepsin D	C-terminus	Primarily Ala124 and Gly132 Additional site Met116	10–13 kDa	Potentially aggregating fragments*	Hossain et al., 2001; Sevlever et al., 2008; McGlinchey et al., 2020
Cathepsins E	Unknown	Unknown	Fragments between 5 and 13 kDa	Unknown	McGlinchey et al., 2020
Cathepsins G	Unknown	Unknown	Most prominent at 10 kDa	Unknown	McGlinchey et al., 2020
Cathepsin K	Spans the whole protein	Monomer: Ser9, Ala27, Ala53, Gly68, Thr75, Glu114	Multiple	Unknown	McGlinchey et al., 2020
	N and C-terminus	Fibrils: Ser9, Gln109, Glu114, and Ser129	10–140, 10–129, 10–114, and 10–109	Potential to enhance aggregation*	McGlinchey et al., 2020
Cathepsin L	Spans the whole protein	Monomer: Met5, Ser9, Ala17, Ala27, Val40, Gly41, His50, Ala53, Gly67, Thr75, and Asn103	1–17, 18–140, 1–103, 104–140, 1–41, 42–140, 1–50, 51–140, 1–53, 54–140, 1–75, 76–140, 6–140, 10–140, 28–140, 1–40, and 68–140	Unknown	McGlinchey et al., 2017
	N and C-terminus	Fibrils: Met5, Gly101, Asn103, Glu114, Asn122, Glu126 and Gln134	1–134, 1–126, 1–122, 1–114, 1–103, 1–101, 6-114	Potential to enhance aggregation*	McGlinchey et al., 2017
Cathepsins S	Unknown	Unknown	10kDa	Unknown	McGlinchey et al., 2020
Cathepsins V	Unknown	Unknown	10 kDa	Unknown	McGlinchey et al., 2020
Cathepsin B	Unknown	Gly14 and Ala90	Several fragments, most prominent at 10 kDa	No effect on seeding capabilities	Tsujimura et al., 2015; McGlinchey et al., 2020
Calpain-1	N-terminus or central region	Ala18, Gly31, Tyr39, Glu57, Gly73, Thr75 and Glu83	Unknown	Controversial *in vitro* results on aggregation potential. *In vivo*, enhances aggregation	Greenbaum et al., 2005; Mishizen-Eberz et al., 2005; Kim H. J. et al., 2006; Dufty et al., 2007; Diepenbroek et al., 2014
	C-terminus	Glu114 and Asn122	Unknown	Potential to enhance aggregation*	Mishizen-Eberz et al., 2005; Diepenbroek et al., 2014

**Huntingtin**					

**Protease**	**Cleavage region**	**Cleavage site**	**Fragments produced**	**Effect on spreading/aggregation**	**References**

MMP-10	C-terminus	Gly402	45 and 55 kDa	Potential to enhance aggregation*	Miller et al., 2010
MMP-14 and -23B	Unknown	Unknown	55 kDa	Potential to enhance aggregation*	Miller et al., 2010
Calpain	C-terminus	Val347 or Ile494	1-347 and 1-494 fragment	Potential to enhance aggregation*	Gafni et al., 2004; Landles et al., 2010
	C-terminus	Ala468-Val470 and Ser535-Val537	45 and 72 kDa	In cells, enhances aggregation	Kim et al., 2001; Gafni and Ellerby, 2002; Lunkes et al., 2002; Kim et al., 2003; Gafni et al., 2004
	C-terminus	Unknown	∼100 kDa	*In vitro and in vivo*, enhances aggregation	Menzies et al., 2015; Weber et al., 2018
Cathepsin B	C-terminus	Unknown	55–60 kDa	Potential to enhance aggregation*	Kim J. et al., 2006
Cathepsin L	C-terminus	Unknown	55–60 kDa	Potential to enhance aggregation*	Kim J. et al., 2006
Cathepsin D	C-terminus	Unknown	45–60 kDa	Potential to enhance aggregation*	Kim J. et al., 2006

**TDP-43**					

**Protease**	**Cleavage region**	**Cleavage site**	**Fragments produced**	**Effect on spreading/aggregation**	**References**

Calpain	Unknown	Leu229, Glu246, Gln286, Gly295, Ala297, Met323	Unknown	Neurotoxic	Yang et al., 2014

**Amyloid beta**					

**Protease**	**Cleavage region**	**Cleavage site**	**Fragments produced**	**Effect on spreading/aggregation**	**References**

Neprilysin	Unknown	Unknown	Unknown	*In vivo*, reduces Aβ peptide levels and plaque burden	Yasojima et al., 2001; Marr et al., 2004; Saido and Leissring, 2012
Neprilysin 2	Unknown	Unknown	Unknown	*In vivo*, decreases Aβ peptide levels	Saido and Leissring, 2012
Insulin-degrading enzyme	Unknown	Unknown	Unknown	Decreases Aβ peptide levels	Farris et al., 2003
MMP-2, MMP-9 and MMP-14, Cathepsin B and Plasmin	Unknown	Unknown	Unknown	Decreases Aβ fibrils	Saido and Leissring, 2012; Hernandez-Guillamon et al., 2015

*Not yet experimentally addressed. Aβ, amyloid-beta; MMP, matrix metalloproteinases; MTBR, microtubule binding region; NAC, non-amyloid component; PHF, paired helical filament.

Thrombin was also found to cleave tau at multiple arginine and lysine residues in the MTBR and proline-rich domains (Arai et al., 2005; Quinn et al., 2018; Zhang et al., 2021). In AD brains, thrombin was found to be upregulated and co-localized with amyloid plaques, microglia and NFTs (Arai et al., 2006). However, PHFs of tau extracted from AD brains were more resistant to thrombin cleavage compared to dephosphorylated PHFs (Arai et al., 2005). The seeding potential of tau fragments produced by thrombin is largely unknown, but tau 125–230 generated by thrombin-mediated cleavage at Gln124-Ala125 was shown to be non-toxic (Garg et al., 2011). This site is also cleaved by calpain-1 and calpain-2 (Garg et al., 2011). However, it is important to note that thrombin-cleaved tau fragments are yet to be identified in tauopathy brains, thereby questioning the pathophysiological relevance of this process.

Several cathepsins have been shown to interact with and modulate tau spreading. As previously mentioned, these proteases are mainly localized to lysosomes, but they can also be secreted in the extracellular space (Boonen et al., 2016; Vidak et al., 2019; Niemeyer et al., 2020). Whilst the majority of data on the action of cathepsins on tau mostly reflects their activity in lysosomes, independent experiments have been conducted either *in vitro* using recombinant proteins or using cathepsin knockout animals. Therefore, a role for extracellular cathepsins cannot be ruled out. Cathepsins B, D, L, and S have all been proposed to cleave tau. Although cathepsin B accumulates in close proximity to NFTs and amyloid plaques (Ii et al., 1993), there is no direct evidence that this protease can cleave tau. In contrast, cathepsin D cleaves recombinant tau at several sites, mostly sparing the MTBR (Kenessey et al., 1997), and as such these fragments could retain the ability to generate higher-order PHFs. At least one of these fragments was produced at neutral pH *in vitro*, suggesting that this cleavage could also occur in the extracellular space (Kenessey et al., 1997). Furthermore, cathepsin D upregulation was detected in an aging *Drosophila* model of tauopathy. The same group observed enhanced tau-induced neurodegeneration in cathepsin D-deficient flies which suggests that this protease may have a neuroprotective effect (Khurana et al., 2010). Cathepsin L has also been found to cleave tau at neutral pH. This activity targets sites within the MTBR, generating several fragments, such as tau 258–360 and tau 258–363, which enhance the aggregation of the full-length protein (García-Sierra et al., 2008; Wang et al., 2009; Zhang et al., 2021). Cathepsin S has been seen to associate with NFTs and its levels are elevated in the brain of AD patients (Lemere et al., 1995; Munger et al., 1995; Nübling et al., 2017). Treatment with cathepsin S *in vitro* yields a distinct cleavage pattern of tau, in which the MTBR seems to remain intact, allowing its association to NFTs. However, this study did not specify whether cleavage occurred at an acidic or neutral pH (Nübling et al., 2017). It is important to note that cathepsin-cleaved tau fragments have yet to be identified in AD brains, highlighting the need for further investigations to unravel the pathophysiological relevance of this protease in AD.

Calpains are proteases mainly found in the cell cytosol (Goll et al., 2003), albeit secretion of calpain has also been observed (Frangié et al., 2006; Letavernier et al., 2012; McDougall et al., 2021), including from isolated rat brain synaptosomes (Pestereva et al., 2021). Calpain has been detected in the CSF, though it remains unclear whether its presence is due to leakage from dying cells (Laske et al., 2015). This dual localization makes investigations on the role of calpain in the context of tau spreading and seeding challenging and as such, the action of extracellular calpains has not been directly investigated as yet. Both calpain-1 and -2 cleave tau (Chesser et al., 2013), and play opposing roles in inducing neurodegeneration (Baudry and Bi, 2016). Calpain-1 activity is significantly upregulated in AD cortical brain tissue from Braak stage III (Kurbatskaya et al., 2016). Much of the interest surrounding the role of calpain in tau pathology has centered on the 17 kDa N-terminal to mid-domain tau fragment (45–230), which is generated through cleavage by calpain-1 at Lys44-Glu45 (Yang and Ksiezak-Reding, 1995) and by calpain-1 (Park and Ferreira, 2005) or -2 (Garg et al., 2011) at Arg230-Thr231. These events have been observed *in vitro*, suggesting calpain-1 and -2 could also exert their activity in the extracellular space, although direct evidence is still lacking. This fragment has been observed in patients affected by AD and other tauopathies, such as progressive supranuclear palsy (PSP) and corticobasal degeneration (CBD) (Ferreira and Bigio, 2011; Garg et al., 2011). However, the specific effect of this tau fragment remains controversial as it was found to induce apoptosis (Park and Ferreira, 2005; Amadoro et al., 2006; Park et al., 2007; Reinecke et al., 2011; Lang et al., 2014) or morphological changes in cell lines and primary neurons (Chen et al., 2021), whereas other groups have observed no such alterations (Garg et al., 2011). Nonetheless, both *Drosophila* and mouse models overexpressing tau 45–230 showed accelerated hippocampal degeneration and synaptic defects (Reinecke et al., 2011; Lang et al., 2014). However, when this 17 kDa fragment was incubated with full-length tau, it significantly lowered aggregate formation, suggesting a possible protective effect (Ferreira and Bigio, 2011). Fascinatingly, aggregation and phosphorylation of full-length tau protected it from calpain cleavage (Ferreira and Bigio, 2011), which is in line with previous data indicating aggregated tau is less susceptible to protease cleavage (Yang and Ksiezak-Reding, 1995; Yang et al., 1997). In a recent study investigating stroke biomarkers, this 17 kDa tau fragment accumulated in primary neurons and their media upon hypoxic treatment, and was decreased by calpain inhibitors (Chen et al., 2021). These findings suggest that calpain-mediated cleavage is specific and can be initiated under different pathological conditions. Similarly, the AD-relevant fragment, tau 26–230, is generated by calpain-1 (Park and Ferreira, 2005) and calpain-2 (Garg et al., 2011) cleavage at Arg230-Thr231. In contrast, Matsumoto and colleagues (Matsumoto et al., 2015) have demonstrated that tau is cleaved *in vitro* by calpain-1 at Arg242-Lys243 producing a 24 kDa C-terminal fragment lacking the N-terminal projection domain (aa 243–441; CTF24). This truncated tau accelerated heparin-induced aggregation and was unable to support microtubule assembly. Furthermore, CTF24 efficiently propagated to other tau-expressing cells, where it displayed higher aggregation and seeding activity than full-length tau (Matsumoto et al., 2015). Interestingly, active calpain-2 co-localizes with tau filaments in AD, Down syndrome and FTD brains (Adamec et al., 2002a,b). A recent study by Cicognola et al. (2020) identified that calpain-2, but not calpain-1, cleaves tau at Lys224, generating an N-terminal fragment previously found to be enriched in CSF in tauopathies (Cicognola et al., 2019). Knockdown of the calpain-2 catalytic subunit gene caused a significant reduction of this N-244 tau fragment in cell-conditioned media (Cicognola et al., 2019). Overall, calpain cleavage appears to promote tau aggregation, thus potentially enhancing its spreading.

## α-Synuclein

α-Synuclein is a small cytosolic protein highly abundant in neurons, and is predominately present as a soluble monomer in physiological conditions (Lee and Trojanowski, 2006). It is composed of 140 amino acids forming three main regions: the N-terminus (aa 1–60) which contains apolipoprotein binding motifs, the central non-amyloid component (NAC) (aa 61–95), which has the propensity to fold into beta sheets, and a negatively charged mostly unstructured C-terminus (aa 96–140) (Stefanis, 2012). The function of α-synuclein remains elusive, although reports on its presynaptic localization and ability to interact with lipids suggest it might have a role in neurotransmitter release (Lee and Trojanowski, 2006; Bernal-Conde et al., 2019). The presence of misfolded, aggregated α-synuclein in Lewy bodies is the molecular hallmark of PD and other neurological conditions termed synucleinopathies, and mutations in its coding gene, *SNCA*, are causative of PD (Stefanis, 2012). α-synuclein was the first pathological protein shown to behave in a manner similar to the prion protein (Karpowicz et al., 2019) and is found extracellularly in the brain and in extracellular fluids, such as CSF (El-Agnaf et al., 2003; Lee et al., 2005). Injection of anti-α-synuclein antibodies in the mouse brain parenchyma halts propagation of α-synuclein pathology (Tran et al., 2014), supporting the possibility of its interneuronal transfer as a free protein. Similar to tau, α-synuclein endocytosis has been shown to occur through interaction with HSPGs and CSPGs (Figures 1Aii,Bii), and it is similarly susceptible to cleavage by extracellular proteases (Table 1).

### Proteoglycans

In C17.2 mouse-derived neural stem cells, internalized α-synuclein fibrils colocalize with HSPGs (Holmes et al., 2013). Similar to tau, α-synuclein endocytosis in cell lines is inhibited by co-application of heparin in a dose-dependent manner (Holmes et al., 2013; Ihse et al., 2017). Heparin acts competitively by binding α-synuclein at sites responsible for its interaction with HSPGs, thus blocking its internalization. However, heparin has been found to be less efficient in blocking internalization of monomeric and oligomeric forms of α-synuclein (Ihse et al., 2017). A neuroblastoma cell line exposed to heparin lyases I, II, and III showed reduced endocytosis of α-synuclein fibrils and similarly, cells lacking enzymes responsible for HPSGs biogenesis fail to internalize α-synuclein (Ihse et al., 2017). Additionally, HSPG sulfation was found to be important, as treatment with chlorate, which inhibits sulfation, reduces α-synuclein fibril internalization (Holmes et al., 2013; Hudák et al., 2019). Further analyses on the sulfation requirements of HSPGs was carried out using differentially sulfated heparin. When applied to C17.2 cells, 2-O, 6-O and *N*-desulphated heparin showed lower efficiency in inhibiting α-synuclein uptake and seeded aggregation compared to standard heparin. Shorter heparin chains are also less efficient in inhibiting α-synuclein uptake and seeding. Accordingly, a CRISPR/Cas9 genetic screen in HEK293 cells identified *EXT1, 2*, and *3*, which mediate the initiation and elongation of the glycosaminoglycan chain in HSPGs, to be involved in α-synuclein uptake and seeding. Similarly, *NDTS1*, which encodes an enzyme responsible for *N*-deacetylation and *N*-sulfation of HSPGs, also appeared to play a role, as its knockout reduced uptake and seeding of α-synuclein. Although the ablation of *HS6ST2*, an enzyme involved in the 2-O sulfation of HSPGs, did not show overt effects on α-synuclein fibril uptake, its overexpression decreased α-synuclein internalization but increased seeding. This suggests a more complex role of 2-O sulfation on α-synuclein spreading (Stopschinski et al., 2018). Zhang et al. (2020) recently analyzed the interaction between α-synuclein and HSPGs by molecular modeling, suggesting that α-synuclein fibrils display more stable binding to HSPGs compared to its monomeric or dimeric forms, possibly explaining the stronger inhibitory effect of heparin on fibril internalization (Zhang et al., 2020).

In a follow up study, treatment of neuroblastoma cells or primary neurons with heparinase failed to inhibit internalization of N-terminal acetylated α-synuclein monomers or fibrils. Since this modification is present at high levels *in vivo* (Burré et al., 2013), this result questions the physiological importance of HSPGs in this process. In contrast, peptide-*N*-glycosidase F (PNGase F), which cleaves complex N-linked glycans from glycoproteins, reduced endocytosis of both fibrillar and monomeric *N*-acetylated α-synuclein but did not affect non-acetylated α-synuclein. Interestingly, acetylated α-synuclein was found to interact with glycans in the absence of their protein core (Birol et al., 2019). Of note, surface membrane proteins as well as secreted proteins and ECM components are heavily glycosylated (Scott and Panin, 2014), creating an ideal multivalent binding environment for pathological α-synuclein. These data indicate that HSPGs are fundamental players in the internalization of α-synuclein fibrils.

CSPGs have also been implicated in α-synuclein aggregation and spreading (Lehri-Boufala et al., 2015; Mehra et al., 2018). Accordingly, incubation of α-synuclein with chondroitin sulfate A and B *in vitro* enhances the formation of aggregates able to enter SH-SY5Y cells (Mehra et al., 2018). Interestingly, chondroitin sulfate, and glycosaminoglycans in general, inhibit cathepsin D, suggesting that the endocytosis of an α-synuclein-chondroitin sulfate complex could induce higher seeding effects due to lysosomal inhibition (Lehri-Boufala et al., 2015). However, co-injection of α-synuclein aggregates with chondroitinase in mice did not change the spread of pathology. On the other hand, degradation of hyaluronan, another major component of the ECM, reduced α-synuclein pathology in the same model, although the mechanism at the basis of this effect is currently unclear (Soria et al., 2020).

### Proteases

It is generally assumed that fragments of α-synuclein must contain the NAC region to propagate pathology. *In vitro* studies using recombinant α-synuclein found that negative charges at the α-synuclein C-terminus counteracts the aggregation propensity of the NAC domain (Izawa et al., 2012). As such, cleavage of the C-terminus by proteases increases aggregation and seeding (Sorrentino et al., 2018, 2020; Chakroun et al., 2020; Sorrentino and Giasson, 2020). Interestingly, α-synuclein carrying PD-linked mutations is more efficiently cleaved at the C-terminus than the wildtype protein (Li et al., 2005).

Conversely, the effect of N-terminal truncation is less clear. Most studies report no change (Luk et al., 2009; Volpicelli-Daley et al., 2011) or a decrease (Luk et al., 2016) in seeded aggregation, even though removal of the first two apolipoprotein binding motifs can lead to an increase (Kessler et al., 2003; Sorrentino and Giasson, 2020). Increased propagation of α-synuclein pathology after brain injection of recombinant α-synuclein lacking the first 10 or 30 residues, compared to full length fibrils has been observed (Terada et al., 2018), suggesting that the N-terminus of α-synuclein can also modulate aggregation. Therefore, the action of extracellular proteases has the potential to both negatively and positively modulate α-synuclein propagation.

Different MMPs such as MMP-1, -2, -3, -9, and -14 cleave α-synuclein, with MMP-3 proven to be the most effective (Levin et al., 2009). *In vitro* tests have shown cleavage at multiple sites at the N-terminus of the NAC (Sung et al., 2005). These cleavage products were observed in the extracellular media of neuroblastoma SK-N-BE cells overexpressing human α-synuclein. MMP-3-cleaved α-synuclein had an elevated propensity to aggregate *in vitro* and displayed increased toxicity compared to full length α-synuclein when applied to SK-N-BE cells (Sung et al., 2005). Interestingly, MMP-3 is elevated in rat brains exposed to 6-hydroxydopamine or 1-methyl-4-phenyl-1,2,3,6-tetrahydropyridine, two classical models of parkinsonism (Sung et al., 2005; Leem et al., 2020). Accordingly, MMP-3 localization in Lewy bodies was detected in the substantia nigra of PD brains (Choi et al., 2011). Choi et al. (2011) investigated the MMP-3-dependent cleavage of α-synuclein harboring mutations associated with PD, and observed that *in vitro*, the A53T mutant is processed more efficiently than the wildtype and A30P mutant proteins, especially at sites 78–79 and 91–93. In contrast, the overexpression of MMP-3 and α-synuclein in COS cells produced lower levels of insoluble α-synuclein aggregates. However, overexpression of the A53T-containing 1–93 α-synuclein fragment in mice induced increased toxicity and formation of Lewy body-like structures in dopaminergic neurons of the substantia nigra, both at the site of injection of the α-synuclein-encoding adeno-associated virus and contralateral side (Choi et al., 2011). These results imply that the 1–93 fragment, which contains the full NAC domain and lacks the C-terminus, is prone to aggregation and spreading. Nevertheless, this is not the only fragment produced by MMP-3 cleavage and the overall effect of MMP-3-cleaved α-synuclein has yet to be conclusively evaluated *in vivo*.

MMP-2 has also been suggested to degrade α-synuclein fibrils. Injection of MMP-2 into animals inoculated with α-synuclein in the cortex and striatum led to reduced levels of insoluble and oligomeric α-synuclein. In addition, human α-synuclein positivity was limited to the injection site in MMP-2 treated animals, suggesting reduced spreading (Oh et al., 2017).

Plasmin is a serine protease that degrades fibrin blood clots, and is also involved in inflammation, collagenase activation and synaptic plasticity (Pang et al., 2004). Application of recombinant plasmin to α-synuclein monomers, oligomers or fibrils resulted in their degradation, including when they harbored several PD-linked mutations, including A53T. Interestingly, α-synuclein found in the media of SH-SY5Y cells was also cleavable by plasmin at different sites spanning the N-terminal domain and the NAC. Using a propagation model utilizing SH-SY5Y cells expressing α-synuclein, co-cultured with the microglia-like cell line BV2 lacking α-synuclein, Kim et al. (2012) showed that exogenous plasmin added to the media could reduce spreading between these two cell types. However, further studies are necessary to confirm the importance of plasmin cleavage on α-synuclein aggregation and spreading *in vivo*.

α-Synuclein has been found to be degraded by neurosin, also called kallikrein 6. Similar to other members of the kallikrein family, neurosin is a secreted trypsin-like serine protease that is activated extracellularly by sequential cleavage (Yoon et al., 2008) and can be found in human CSF (Diamandis et al., 2000). *In vitro* treatment of recombinant α-synuclein with neurosin generates several fragments by cleavage within and in the proximity of the NAC domain. Neurosin digestion was found to inhibit α-synuclein aggregation *in vitro* (Iwata et al., 2003), and treatment of α-synuclein oligomers with neurosin induced their almost complete degradation (Spencer et al., 2013). Pro-aggregation variants such as α-synuclein phosphorylated at Ser129 (Kasai et al., 2008) or carrying the PD-linked mutations A30P, A53T or E46K showed reduced cleavage by neurosin (Iwata et al., 2003; Kasai et al., 2008; Spencer et al., 2013). Of note, neurosin levels are lower in brains of patients affected by dementia with Lewy bodies and in α-synuclein transgenic mouse models (Spencer et al., 2013). Furthermore, overexpression of neurosin in HEK293 cells or in cortical neurons induced degradation of α-synuclein in the culture media, whereas primary neurons from neurosin knockout mice showed increased α-synuclein internalization and aggregation (Tatebe et al., 2010; Pampalakis et al., 2017). In addition, neurosin can also activate proMMP-2 (Pampalakis et al., 2017) and an unidentified extracellular protease (Ximerakis et al., 2014), further promoting α-synuclein degradation. Although it is unclear whether neurosin acts directly or through activation of a downstream protease, its activity appears to strongly reduce both aggregated and monomeric forms of α-synuclein.

Cathepsins B, D, E, G, K, L, S and V have been shown to cleave α-synuclein, both in its monomeric and fibrillar forms. In particular, both cathepsins L and K were shown to completely ablate α-synuclein fibrils, whereas cathepsins B, D, E, G, S and V generate small α-synuclein fragments (McGlinchey and Lee, 2015; McGlinchey et al., 2017, 2020). Importantly, degradation of α-synuclein by cathepsins L and K was achieved upon long incubation (16 h) and at an acidic pH, which would be typically found in lysosomes. Shorter incubation times or treatment at a neutral pH mimicking that of the extracellular space, generated fragments truncated at the N- and C-termini (McGlinchey et al., 2017, 2020). As discussed above, these fragments display an increased aggregation propensity, albeit this was not tested experimentally. Cathepsin B was found to cleave both α-synuclein monomers and fibrils *in vitro* upon incubation at low pH. It is thus unclear whether this activity could be retained at neutral pH in the extracellular space (McGlinchey and Lee, 2015; Tsujimura et al., 2015). C-terminal cleaved fragments were also observed upon addition of recombinant cathepsin B to lysates of 3D5 cells expressing human α-synuclein, or to mouse and human brain extracts (Sevlever et al., 2008; Tsujimura et al., 2015). Cathepsin D was also found to cleave recombinant α-synuclein *in vitro* at residues 116, 124 and 132, thus potentially generating aggregating fragments (Hossain et al., 2001; Sevlever et al., 2008). Since the proteolytic activity of both cathepsin B and D on α-synuclein was only observed at acidic pH, it is questionable whether these events occur in the extracellular space. However, cathepsin D knockout mice display insoluble α-synuclein in brain extracts, even in the absence of overexpression. A similar result was observed in human post-mortem brains affected by mutations in the *CTSD* gene (Cullen et al., 2009). Accordingly, knockdown of the *C. elegans Ctsd* ortholog caused increased aggregation of overexpressed human α-synuclein, whereas overexpression of *Ctsd* in α-synuclein expressing worms increased survival of DA neurons, an effect that was not present upon expression of cathepsins B and L (Qiao et al., 2008).

The effects of calpain-1 on α-synuclein have been studied in detail. However, similar to the action of calpain on tau, its activity on α-synuclein has been assumed to occur intracellularly. Calpain cleaves monomeric α-synuclein mostly within the N-terminal or central region (Mishizen-Eberz et al., 2003; Greenbaum et al., 2005; Kim H. J. et al., 2006; Diepenbroek et al., 2014). The aggregation potential of monomeric α-synuclein treated with calpain is still controversial (Mishizen-Eberz et al., 2005; Dufty et al., 2007). *In vitro*, calpain-1 cleaves both wildtype and PD mutant forms of fibrillar α-synuclein within the C-terminus (Mishizen-Eberz et al., 2003; Diepenbroek et al., 2014), which may increase the aggregating potential of α-synuclein. Diepenbroek and colleagues evaluated the role of calpain *in vivo* by crossing mice expressing human α-synuclein carrying the A30P mutation with a calpastatin knockout or overexpressing mouse model. Overexpression of calpastatin, which acts as a calpain inhibitor, reduced α-synuclein aggregates in the brain, whereas calpastatin knockout mice showed the opposite effect (Diepenbroek et al., 2014). Similar findings were obtained by treating mice expressing human wildtype α-synuclein with the calpain inhibitors gabadur and neurodur. This treatment decreased insoluble α-synuclein aggregates with a concomitant reduction in gliosis, neurodegeneration and hyperactivity (Hassen et al., 2018). Therefore, calpain processing of α-synuclein appears to increase its aggregation potential.

## Huntingtin

Huntingtin is a large 384 kDa protein that contains an N-terminal region, which functions as a nuclear export signal, followed by a stretch of glutamines that contains between 9 and 35 residues in healthy subjects. Higher numbers of CAG repeats, which encode for these glutamines, in the huntingtin gene are associated with aggregation and HD. The remaining portion of this protein is far less characterized, although it presents several HEAT repeats that are important for protein–protein interactions. Huntingtin is involved in several functions from vesicular trafficking to translation and autophagy (Saudou and Humbert, 2016). The neuronal spread of huntingtin pathology has been described; however, the evidence is less definitive compared to other proteins discussed in this review (Pecho-Vrieseling et al., 2014). Aggregates of recombinant polyQ peptides are internalized and induce further aggregation in HEK293 cells (Ren et al., 2009). Moreover, huntingtin aggregates extracted from the brain of R6/2 mice, which overexpress human huntingtin exon1 carrying ∼115 CAG repeats, were able to increase aggregation of overexpressed huntingtin in Neuro2a cells (Nekooki-Machida et al., 2009). However, in HD patients that received fetal neural allografts, aggregated huntingtin was found in the graft region in the extracellular space but not in neurons, arguing against the ability of aggregated huntingtin to spread between cells *in vivo* (Cicchetti et al., 2014). In this light, further research is required to confirm the relevance of huntingtin spreading in the progression of HD.

Aggregation of huntingtin is driven by polyQ expansion and, as such, this region needs to be retained for huntingtin to act as a seed. In addition, N-terminal fragments of huntingtin have been shown to be more toxic than the full-length protein. For example, shorter versions of huntingtin terminating at residue 145 or 650 are more aggregation-prone in a polyQ length-dependent manner and induce increased sensitivity to oxidative stress in primary neurons compared to the full-length protein. Interestingly, these fragments recruit endogenous huntingtin into aggregates (Martindale et al., 1998). Further studies confirmed that the shorter the huntingtin fragment, the more aggregation prone it is, as long as the polyQ region is retained (Hackam et al., 1998). This observation therefore suggests that extracellular proteolytic cleavage could modulate disease progression (Graham et al., 2006; Ratovitski et al., 2011; O’Brien et al., 2015). C-terminal fragments have been less extensively studied. Work from El-Daher et al. (2015) found that the 587–3144 fragment could cause toxicity on its own, but had a protective effect against fragment 1–167–mediated toxicity, whereas it potentiates 1–586 toxicity in both striatal cells and *Drosophila*.

### Proteases

Truncated huntingtin species are generated through cleavage by different proteases that can be found in the extracellular space such as MMPs, calpains and cathepsins (Table 1). MMP-10, -14 and -23B were identified in an shRNA-based knockdown screen in HEK293 cells looking for a reduction in huntingtin fragments. Knockdown or pharmacological inhibition of these MMPs reduced toxicity in a striatal cell line expressing huntingtin with 111 glutamines, as well as in *Drosophila* lines expressing the first 336 amino acids of the human huntingtin protein with 128 glutamines. MMP-10 was confirmed to cleave recombinant huntingtin both *in vitro* and in cell lysates, mostly producing a 45 kDa fragment which could also be observed in brain extracts from HD patients. MMP-10 and -14 activity was increased in a striatal cell line expressing huntingtin with 111 glutamines compared to a non-pathogenic variant with only 7 (Miller et al., 2010). Furthermore, increased MMP-2, -3 and -10 activity was observed in neural stem cells from HD patients compared with their isogenic lines, where the polyQ expansion was corrected. In contrast to this, the activities of MMP-9 and -14 were found to be reduced in HD (Naphade et al., 2017).

Calpain also mediates proteolytic processing of huntingtin. Application of recombinant calpain-1 to recombinant huntingtin or to mouse brain extracts caused the appearance of fragments with molecular weights between 45 and 72 kDa, which could also be observed in post-mortem brain samples of both healthy and HD-affected subjects (Kim et al., 2001, 2003; Gafni and Ellerby, 2002; Lunkes et al., 2002). However, high-polyQ huntingtin appeared to be more resistant to calpain cleavage compared to lower repeat mutants *in vitro* (Gafni and Ellerby, 2002). Calpain-resistant variants showed reduced aggregation and toxicity in HEK293T cells compared to calpain-cleavable huntingtin (Gafni et al., 2004). In addition, calpain-derived fragments have been observed both in mouse models expressing human huntingtin with 150 polyQ (Gafni et al., 2004; Landles et al., 2010), and in post-mortem caudate samples from HD patients which also presented increased calpain levels (Gafni and Ellerby, 2002; Gafni et al., 2004). These results were confirmed in *Drosophila*, where knocking out calpain reverted the toxic effects of huntingtin overexpression, although this effect appeared to be due to the intracellular activity of calpain, since a double knockout for calpain and the autophagic protein Atg8 failed to show the same rescue effects (Menzies et al., 2015). Furthermore, crossing calpastatin knockout mice with HD mice with 111 polyQ showed increased production of huntingtin N-terminal fragments and subsequent aggregation (Weber et al., 2018). Similar to tau and α-synuclein, a direct demonstration of calpain-mediated cleavage of huntingtin in the extracellular space is still lacking.

Cathepsins also cleave huntingtin. In particular, cathepsins B, D and L were found to generate two 55–60 kDa fragments when incubated with striatal cell lysates. Interestingly, cathepsin D only generated an N-terminal fragment from wildtype huntingtin, whereas up to four different fragments were detected when huntingtin containing 100 polyQ was exposed to cathepsin D. This cleavage was carried out at a neutral pH, suggesting that it could occur in the extracellular space (Kim J. et al., 2006).

## TDP-43

Transactive response (TAR) DNA binding protein 43 (TDP-43) is a protein involved in transcriptional regulation and processing of thousands of different RNAs (Prasad et al., 2019). Under physiological conditions, it is localized in the nucleus but redistributes to the cytosol in several neuropathologies. TDP-43 aggregation is a hallmark of FTD and amyotrophic lateral sclerosis (ALS) and mutations in its coding gene are causative for these diseases (Chhangani et al., 2021). Evidence of its prion-like activity has also been described. For example, injection of brain extracts from FTD patients induced formation of aggregates in neuronal cell lines (Nonaka et al., 2013) and mouse models (Porta et al., 2018; Peng et al., 2020). TDP-43 seeds were also able to transfer between cultured neurons grown in microfluidic devices both as a free protein and inside exosomes (Feiler et al., 2015).

TDP-43 is composed of an N-terminal domain, two RNA recognition motifs and a C-terminal domain. Cryo-electron microscopy studies have identified the core of TDP-43 aggregates to be formed of residues 282–360 (Cao et al., 2019; Arseni et al., 2021). Several TDP-43 fragments have been observed in human samples from ALS-FTD patients (Smethurst et al., 2016) and C-terminal fragments appear to retain aggregation properties (Igaz et al., 2009; Nonaka et al., 2009; Furukawa et al., 2011; Shimonaka et al., 2016). Several studies suggest that a 25 kDa C-terminal fragment, possibly generated by cleavage at residue 216, (Caccamo et al., 2015) promotes seeded aggregation. This fragment is more abundant in the brain than in spinal cord samples isolated from the same FTD patient and, in accordance, brain extracts showed increased seeding capabilities compared to spinal cord extracts (Smethurst et al., 2016). However, other studies have found that expression of this C-terminal fragment in mice does not cause aggregate formation and drives limited behavioral defects (Caccamo et al., 2012, 2015; Akamatsu et al., 2013; Dayton et al., 2013; Walker et al., 2015; Porta et al., 2018). Other C-terminal fragments have also been identified (CT35kDa and CT27kDa), but their aggregation and seeding properties are still controversial (Zhang et al., 2009; Suzuki et al., 2011). In contrast, a recent study showed that injection of aggregates of an N-terminal 1–265 fragment was sufficient to induce formation of TDP-43-positive cytosolic stress granules and cause neuronal death (Pirie et al., 2021).

To the best of our knowledge, there is no evidence directly linking ECM core components with TDP-43 spreading. However, calpains have been found to process TDP-43, and as mentioned for other prion-like aggregates, it is possible that this protease could modify the seeding properties of TDP-43 in the extracellular milieu. Calpains cleave TDP-43 *in vitro* at multiple sites in mouse and rat brain extracts (Table 1). These fragments retained toxicity when applied to cultured primary neurons. Similar fragments were observed in mouse models of traumatic brain injury (TBI) and in the CSF of TBI patients (Yang et al., 2014). Interestingly, phosphorylated TDP-43 is resistant to calpain cleavage *in vitro* (Yamashita et al., 2016), whereas the A315T and M337V TDP-43 mutants showed increased processing (Yamashita et al., 2012). TDP-43 fragments corresponding to calpain cleavage events have been observed in extracts from the motor cortex and spinal cord of FTD-ALS patients with a concomitant increase in activated calpain-1 and -2. Interestingly, both fragments and activated calpain were higher in spinal cord samples compared to the motor cortex (Yamashita et al., 2012).

## Amyloid Beta

Amyloid precursor protein (APP) is an integral transmembrane protein consisting of a large extracellular domain, a transmembrane domain and a short intracellular tail (Chen et al., 2017). APP is widely expressed in neuronal and non-neuronal cells, and its physiological function is still unclear, although it is believed to function as a cell adhesion molecule (Müller et al., 2017). APP is sequentially cleaved by β- and γ-secretase to produce Aβ peptides (Aβ40 and Aβ42), which form the core of amyloid plaques found in the brain of AD patients (Hardy and Selkoe, 2002; Selkoe, 2002; Haass et al., 2012). In neuronal cells, APP is predominantly found in the secretory pathway, from where it is trafficked to the axon termini and dendrites. Once APP reaches the plasma membrane, it is quickly internalized, leading to only a small fraction being present on the neuronal surface (Haass et al., 2012). Accordingly, Aβ production is likely to occur in the endocytic pathway, since inhibiting the internalization of cell surface APP leads to a significant decrease in Aβ production (Koo and Squazzo, 1994; Koo et al., 1996). The formation of extracellular Aβ aggregates is discussed below, since the involvement of ECM components in this process has been more extensively studied.

Very little is known about the precise molecular mechanisms triggering the conversion of soluble Aβ peptides to oligomers, protofibrils, amyloid fibrils and plaques in the brain (Friesen and Meyer-Luehmann, 2019). Intraneuronal Aβ42 accumulation precedes the deposition of extracellular plaques and has been observed in multivesicular bodies, endosomes and along microtubules (Takahashi et al., 2002, 2004). These intraneuronal Aβ42 clusters are enriched in pyramidal neurons of the hippocampus and entorhinal cortex, both of which are particularly vulnerable to pathology (Gouras et al., 2000). Most Aβ peptides are released from distal axons and synapses, and spread to synaptically connected regions (Friesen and Meyer-Luehmann, 2019); however, no *in vivo* study has demonstrated the transport and spread of Aβ and the roles that ECM might play. Domert et al. (2014) have shown that oligomeric Aβ is transferred between connecting, differentiated SH-SY5Y cells, and have postulated that this is due to impaired degradation of these. In addition, the cellular mechanism(s) leading to plaque spreading is also not fully elucidated. Thus, many fundamental questions remain about Aβ dynamics *in vivo* and its deposition in plaques in the AD brain.

### Proteoglycans

A class of extracellular proteins which is found to play extensive roles in Aβ uptake, aggregation and deposition is the HSPG family (Figures 1Aiii,Biii). Amyloid plaques were first found to contain HSPGs by Snow and colleagues (Snow et al., 1988), followed by the observation that there is a direct interaction between HSPGs and both APP and Aβ (Narindrasorasak et al., 1991; Brunden et al., 1993). Cell lines deficient in HS biosynthesis, or treated with heparin, show a decrease in the binding of Aβ peptides to the cell membrane and subsequent internalization (Kanekiyo et al., 2011). An AD mouse model with a neuron-specific impairment in HS biosynthesis exhibited decreased plaque burden and Aβ oligomerization (Liu et al., 2016). Interestingly, the low-density lipoprotein-related receptor (LRP1)-dependent endocytosis of Aβ42 relies on HSPGs, since the addition of heparin abrogated the increase in Aβ42 uptake observed by the overexpression of an LRP1 mini-receptor (Kanekiyo et al., 2011). In conjunction with this, the Bu group has observed that ApoE-immunoisolated extracellular vesicles, derived from astrocytes, suppress Aβ binding and uptake in mouse cortical neurons, an effect abrogated by heparin (Fu et al., 2016). These findings suggest that HSPGs are involved in Aβ42 internalization. Although mostly localized at the plasma membrane, syndecans and glypican-1 show extensive co-localization with extracellular amyloid plaques in the brain, with the neuron-specific syndecan-3 being most effective at increasing Aβ42 uptake (van Horssen et al., 2002). By virtue of their HS chains, syndecan-3 and -4 also trigger formation of Aβ42 fibrillar assemblies, underscoring their relevance in plaque formation (Letoha et al., 2019). Glypican-1 interacts with higher-order Aβ structures and reduces SH-SY5Y viability when overexpressing APP (Watanabe et al., 2004). However, its function in plaque seeding or aggregation is currently not known.

Apart from their roles in mediating cell surface binding and endocytosis of APP/Aβ, HSPGs, along with CSPGs, also play a role in clearance and degradation of Aβ peptides (Gupta-Bansal et al., 1995). While post-mortem AD brain samples contain higher than normal levels of HSPGs, clearance of soluble Aβ in the mouse hippocampus is increased upon HSPG depletion (Liu et al., 2016). It is currently postulated that HSPGs function to promote Aβ fibrillation or act as protective chaperones, thus inhibiting Aβ degradation (van Horssen et al., 2003). Both agrin and perlecan, like other HSPGs, bind to Aβ40 fibrils through glycosaminoglycan side chains, protecting them from degradation and accelerating the fibrillation of monomeric Aβ40 (Castillo et al., 1997; Cotman et al., 2000). This role of agrin was recapitulated in an AD mouse model crossed with a conditional *Agrn* KO line where deletion was driven by an endothelial cell-specific promoter. These mice exhibited increased Aβ generation and deposition (Rauch et al., 2011). However, this effect was not observed when neuronal agrin was deleted, pointing to a non-cell autonomous effect on plaque formation.

### Proteases

Proteolytic degradation contributes to the regulation of extracellular Aβ levels and deposition in amyloid plaques. Several extracellular proteases have been implicated in the degradation of Aβ40/42, including neprilysin (NEP) and the insulin-degrading enzyme (IDE) (Table 1; Saido and Leissring, 2012).

Neprilysin is a zinc metalloprotease with broad substrate specificity and cell surface localization. Demonstrating an inverse correlation with age, it is reported to cleave only Aβ monomers along the axon and at synaptic sites, and has negligible enzymatic activity for higher-order Aβ structures (Fukami et al., 2002; Saido and Leissring, 2012). Consistent with its role in the reduction of Aβ42 levels, brain tissues of AD patients exhibit a decrease in NEP expression in AD-vulnerable areas, including the hippocampus, cortex and temporal gyrus (Yasojima et al., 2001). The genetic deletion of NEP or its pharmacological inhibition results in a two-fold increase in Aβ levels in the brain as well as increased hippocampal plaque burden (Marr et al., 2004; Farris et al., 2007). Conversely, overexpression of neuronal NEP results in reduced Aβ levels and deposition, along with a reduction in associated pathology (Leissring et al., 2003). Additionally, its homolog NEP2 also contributes to decreasing Aβ levels (Saido and Leissring, 2012).

The zinc metalloprotease IDE also displays a broad distribution in the extracellular space, cytosol and mitochondria. Similar to NEP, IDE catalyzes the degradation of monomeric Aβ in the brain and its ablation causes an increase in Aβ levels in primary neurons as well as *in vivo* (Farris et al., 2003). Other extracellular proteases processing Aβ include MMP-2, MMP-9, and MMP-14, cathepsin B and plasmin (Saido and Leissring, 2012; Hernandez-Guillamon et al., 2015). Interestingly, these enzymes are capable of catabolizing Aβ fibrils, in contrast to NEP and IDE (Saido and Leissring, 2012). Their investigation *in vivo* remains limited, though cathepsin B has been observed in amyloid plaques (Mueller-Steiner et al., 2006).

### Other Extracellular Matrix Components

In contrast to HSPGs and extracellular proteases, the role of ECM core proteins in Aβ production, seeding or plaque deposition is less clear. Several ECM proteins, including laminin, collagens and fibronectin co-localize with senile plaques in AD brains (Perlmutter et al., 1991). Proteomic profiling of AD hippocampi has revealed an upregulation of several ECM proteins during all disease stages (Hondius et al., 2016). However, whether these observations are causative, or correlative of amyloid formation, is currently unknown.

Laminin1 binds to soluble Aβ40 through its α-chain and inhibits its fibrillogenesis in a time- and dose-dependent manner, thus influencing the survival of cortical neurons (Drouet et al., 1999). While it increases the amyloidogenic fragment production, laminin1 also promotes Aβ40 depolymerization when incubated with pre-formed fibrils *in vitro* (Bronfman et al., 1996; Castillo et al., 2000).

Total collagen levels are upregulated in AD; however, the roles of individual isoforms are currently unclear. The Mucke group has shown that synthetic Aβ42 oligomers induce the transcription of the *Col6a1* gene in hippocampal and cortical neurons and that collagen VI decreases the interaction of Aβ42 oligomers with the neuronal cell surface, ultimately leading to a reduction in Aβ42 neurotoxicity (Cheng et al., 2009).

In addition, when the 7PA2 cell model of AD is treated with peptides derived from elastin, it shows an elevation in Aβ40 and Aβ42 production. This effect was dependent on the length of the individual peptides, since longer peptides were more potent in Aβ generation and led to AD-related behavior in mice. Significantly, these changes were pinned down to an increased expression of β- and γ-secretase mRNAs (Ma et al., 2019).

## Concluding Remarks

In this review we have presented evidence supporting the role of different ECM components in modulating the spread and aggregation of pathological, misfolded proteins. Although the available evidence remains limited, predominately due to the complexity of the ECM and its partial reconstitution observed in cell cultures, available data provide the rationale for further exploration of this area of research. In addition to the proteins discussed in this review, other proteins have been shown to possess the ability of transcellular spread, such as superoxide dismutase 1 (SOD1) (Ayers et al., 2014) and C9orf72 (Westergard et al., 2016) in ALS-FTD. However, to the best of our knowledge, we could not identify studies exploring the contribution of ECM components in mutant SOD1 and C9orf72 spreading.

The findings presented here highlight a high level of overlap between pathways modulating aggregation properties and/or propensity of spreading that affect most of these protein aggregates. In particular, proteoglycans and proteases are shared players in several of these diseases. HSPGs appear to participate in several steps of the spreading process of tau, α-synuclein and Aβ (Figures 1A,B), as well as the prion protein (Horonchik et al., 2005). Furthermore, direct binding to HSPGs has been shown for all three proteins. As mentioned above, HSPGs represent a heterogenous class of molecules. The presence of HSPGs on both the plasma membrane and in the extracellular space further complicates the experimental dissection of the underlying mechanism. Plasma membrane-exposed HSPGs have been clearly linked to internalization of aggregation-prone forms of tau and α-synuclein, a property shared with the prion protein (Horonchik et al., 2005). This suggests that HSPGs represent a common node for the endocytosis of pathological protein aggregates. As discussed above, the interaction appears to be mediated by the glycan groups, rather than the protein core, the contribution of which remains unclear. As such, it is likely that glycans may have some predisposition for binding to aggregated proteins, although the biochemical determinants of these interactions need to be further elucidated. In addition, heparin is routinely used to induce aggregation of recombinant tau and α-synuclein *in vitro* (Goedert et al., 1996; Cohlberg et al., 2002). This points to a potential role of extracellular HSPGs in inducing aggregation of these proteins, thus facilitating their internalization and as such, favoring the spread of pathology.

However, exogenous application of heparin can inhibit internalization of protein aggregates. This suggests that extracellular HSPGs could also act in a similar manner. However, the only study that has directly addressed extracellular HSPGs in this context has demonstrated that agrin promotes aggregation of Aβ and protects aggregates from degradation (Cotman et al., 2000). A similar function has also been proposed for agrin on tau, given the presence of agrin in NFTs (Verbeek et al., 1999). This multi-pronged action of HSPGs on several steps of the spreading process complicates predictions as to whether HSPGs could be targeted to slow down disease progression. Of note, chronic application of heparin mimetics failed to show significant effects on tau pathology in mice (Stopschinski et al., 2020). Interestingly, a role of HSPGs in the internalization of huntingtin aggregates was excluded (Holmes et al., 2013), suggesting a certain level of specificity. An in-depth characterization of this process is therefore required to understand the role of each HSPG species on the different steps of spreading, and to identify the biochemical determinants driving each of these effects. This could potentially lead to the identification of an endogenous HSPG, the levels of which could be modulated to reduce spreading. Alternatively, synthetic molecules that mimic HSPG protective effects could also be designed. Other ECM components, such as CSPGs, laminin, collagen and elastin, have been found to participate in the spreading of protein aggregates (Figure 1), but their role remains less characterized.

Proteases have emerged as major regulators of prion-like pathological spreading. In addition to a general clearing effect on aggregates, specific proteases can process monomers or oligomers of pathological proteins, generating fragments which display altered aggregation propensity (Table 1). Less is known about how these fragments behave with regard to their internalization properties. This area should be further investigated to conclusively establish the contribution of these proteases to the spreading of protein aggregates. MMPs are active against tau, α-synuclein, huntingtin and Aβ, with each MMP performing distinctive roles. MMP-3, for example, has been shown to reduce the propensity of tau to aggregate, whereas it was suggested to increase α-synuclein aggregation. However, MMP-3 is elevated in AD and PD models (Sung et al., 2005; Leem et al., 2020; Pentz et al., 2021) and in patients (Stomrud et al., 2010; Choi et al., 2011; Hanzel et al., 2014). This suggests that MMP-3 expression might be increased in an attempt to reduce pathology, however with opposite outcomes on tau and α-synuclein. Similarly, MMP-9 levels are increased in AD brains (Hernandes-Alejandro et al., 2020), again as a potential compensatory measure to reduce both Aβ and tau pathology. However, not only does MMP-9 degrade Aβ plaques (Hernandez-Guillamon et al., 2015), but it also induces the production of tau fragments with enhanced aggregation properties (Nübling et al., 2012). Conversely, MMP-2 reduces α-synuclein pathology and Aβ plaque burden *in vivo*, but was suggested to increase tau aggregation. Similar to other MMPs, its levels are increased in AD (Terni and Ferrer, 2015). In contrast, MMP-2 is reduced in synucleinopathies (Lorenzl et al., 2002), suggesting its causal role in these diseases. With this exception, these observations indicate that MMPs are upregulated in response to pathology, possibly in an attempt to degrade protein aggregates. However, in several cases, protease activity has proven detrimental, potentiating the aggregation of seeds.

Cathepsins and calpains are key intracellular proteases, the roles of which have been poorly characterized in the extracellular space. This makes the interpretation of the data difficult, as most studies do not specifically investigate whether their action is solely exerted intracellularly, or also extracellularly. Therefore, future experiments should be carried out to clarify whether these proteases would retain activity against proteopathic proteins in the extracellular compartment. Despite this, cathepsins seem to have a protective role by reducing the aggregation of tau, Aβ and α-synuclein. As described above, several cathepsins appear to be upregulated in AD suggesting that lysosomal activity is increased in an attempt to reduce the aggregate load. Whether this reflects an increase of extracellular cathepsin is still unclear. In contrast, risk variants in cathepsin B and D genes were identified in synucleinopathies (Robak et al., 2017; Blauwendraat et al., 2020), which support the involvement of these proteases in the mechanism of disease. The role of these proteases in HD and TDP-43 pathology has not yet been investigated in sufficient detail. The well-defined role of cathepsins in lysosomal degradation, in addition to their presence in the extracellular space, makes them an interesting pharmacological target to modulate protein aggregation and spreading in a two-pronged manner. Conversely, calpain cleavage was suggested to promote aggregation of α-synuclein, tau, TDP-43 and huntingtin, with increased levels of calpain detected in related pathologies. This strongly suggests that increased calpain activation could be a common pathological mechanism in neurodegenerative disorders, thus making it a promising therapeutic target. Further research should therefore be carried out to characterize the extracellular proteases involved in the proteolytic processing of prion-like proteins during the prodromic and early symptomatic phases of disease.

The findings presented in this review support the need to clarify the contributions of ECM components in neurodegenerative diseases. To do so, research should include *in vivo* experiments in animal models of neurodegeneration, which retain the complex ECM web in its native state, and using culture systems better recapitulating this aspect. For example, three-dimensional (3D) organoids generated from human induced pluripotent stem cells (hIPSCs) may allow easier access to study the ECM compared to animal models, albeit maintaining a greater complexity than 2D cultures (Cho et al., 2021). This would not only help the discovery of novel roles for ECM components but could also drive the development of new therapeutic strategies for neurodegenerative diseases. Although these therapies would not directly target initial pathological events, such as the generation of misfolded proteins, they could halt their spreading between brain regions, therefore slowing down disease progression. In addition, given their extracellular localization, these proteins have the advantage of being more easily targetable and their modulation might have reduced side effects as they would not directly affect neuronal and glial cell function. As such, increased efforts in exploring the role of ECM components in modulating the spreading of pathological protein aggregates should be strongly encouraged.

## Author Contributions

EM and GS conceptualized the manuscript. EM, SSt, and SSu wrote the manuscript. EM, SSt, SSu, JV, and GS reviewed and edited the manuscript. GS, JV, and EM acquired funding. All authors contributed to the article and approved the submitted version.

## Conflict of Interest

The authors declare that the research was conducted in the absence of any commercial or financial relationships that could be construed as a potential conflict of interest.

## Publisher’s Note

All claims expressed in this article are solely those of the authors and do not necessarily represent those of their affiliated organizations, or those of the publisher, the editors and the reviewers. Any product that may be evaluated in this article, or claim that may be made by its manufacturer, is not guaranteed or endorsed by the publisher.
